# Prestroke and Poststroke Antithrombotic Therapy in Patients With Atrial Fibrillation: Results From a Nationwide Cohort

**DOI:** 10.1001/jamanetworkopen.2018.0171

**Published:** 2018-05-18

**Authors:** Anna Gundlund, Ying Xian, Eric D. Peterson, Jawad H. Butt, Kasper Gadsbøll, Jonas Bjerring Olesen, Lars Køber, Christian Torp-Pedersen, Gunnar H. Gislason, Emil Loldrup Fosbøl

**Affiliations:** 1 Department of Cardiology, Gentofte Hospital, Hellerup, Denmark; 2 Duke Clinical Research Institute, Durham, North Carolina; 3 Department of Cardiology, University Hospital of Copenhagen, Copenhagen, Denmark; 4 Department of Health Science and Technology, Aalborg University, Aalborg, Denmark; 5 Department of Cardiology, Aalborg University Hospital, Aalborg, Denmark; 6 Department of Epidemiology, Aalborg University Hospital, Aalborg, Denmark; 7 Department of Biostatistics, Aalborg University Hospital, Aalborg, Denmark; 8 Danish Heart Foundation, Copenhagen, Denmark; 9 National Institute of Public Health, University of Southern Denmark, Odense, Denmark

## Abstract

**Importance:**

Antithrombotic therapies are effective in both primary and secondary stroke prophylaxis in high-risk patients with atrial fibrillation (AF), but they are often underused in community practice.

**Objective:**

To examine prestroke and poststroke antithrombotic treatment patterns and long-term outcomes in patients with AF presenting with ischemic stroke.

**Design, Setting, and Participants:**

A retrospective cohort study of Danish patients with AF, with a prestroke CHA_2_DS_2_-VASc score of 1 or higher for men and 2 or higher for women, and presenting with ischemic stroke was conducted from January 1, 2004, to January 31, 2017. Data on hospital admission, prescription fillings, and vital status were assessed using several Danish nationwide registries.

**Exposures:**

Patients who survived 100 days after discharge were divided into 3 groups according to poststroke antithrombotic therapy: oral anticoagulation (OAC) therapy, antiplatelet therapy alone, or no antithrombotic therapy.

**Main Outcomes and Measures:**

Long-term outcomes (thromboembolic events and bleeding complications) were examined using multivariable Cox regression analyses across the 3 groups.

**Results:**

Among 30 626 patients with AF admitted with ischemic stroke, 11 139 patients (36.3%) received OAC therapy (44.3% female; median age, 79 years [interquartile range, 73-85 years]), 11 874 (38.8%) received antiplatelet therapy alone (55.0% female; median age, 82 years [interquartile range, 75-88 years]), and 7613 (24.9%) received no antithrombotic therapy before stroke (53.8% female; median age, 80 years [interquartile range, 71-86 years]). Following stroke, 31.3% of those receiving antiplatelet therapy alone and 43.7% of those receiving no antithrombotic therapy before stroke shifted to OAC therapy. Yet, 37.5% of patients with stroke did not receive OAC therapy following stroke. However, OAC treatment rates increased over time. During a maximum of 10 years of follow-up, 17.5%, 21.2%, and 21.5% experienced a new thromboembolic event and 72.7%, 86.4%, and 86.2% died among those treated with OAC therapy, antiplatelet therapy, or no antithrombotic therapy, respectively. Poststroke OAC therapy was associated with lower risk of recurrent thromboembolic events (adjusted hazard ratio, 0.81; 95% CI, 0.73-0.89) and no significant difference in bleeding complications (adjusted hazard ratio, 0.97; 95% CI, 0.86-1.10), compared with no poststroke antithrombotic therapy. In contrast, there were no significant differences for those treated with poststroke antiplatelet therapy and no antithrombotic therapy.

**Conclusions and Relevance:**

Patients with AF receiving poststroke OAC therapy had lower risk of recurrent thromboembolic events. Our findings suggest a substantial opportunity for improving primary and secondary stroke prophylaxis in high-risk patients with AF.

## Key Points

**Question:**

Is oral anticoagulation therapy used and effective for secondary stroke prophylaxis in patients with atrial fibrillation?

**Findings:**

In this cohort study of 30 626 intermediate- to high-risk patients with atrial fibrillation having an ischemic stroke, 36.3% received oral anticoagulation therapy before their stroke, and 52.5% received oral anticoagulation therapy after their stroke. Oral anticoagulation therapy was associated with a statistically significant reduction in thromboembolic risk.

**Meaning:**

There exists a major potential for optimization of both primary and secondary stroke prophylaxis in patients with atrial fibrillation.

## Teaser

This cohort study uses Danish national registry data to describe use of antithrombotic therapy before and after ischemic stroke in patients with atrial fibrillation, and to investigate associations between use of antithrombotic therapy and long-term recurrent embolism and bleeding.

## Introduction

Patients with atrial fibrillation (AF) have a 5-fold increased risk of ischemic stroke and 20% to 30% of all strokes are generally attributed to AF.^1,2,3,4^ Oral anticoagulation (OAC) therapy with either vitamin K antagonists or non–vitamin K antagonist oral anticoagulants reduces this risk by more than 60% and is recommended as both primary and secondary stroke prophylaxis in patients with AF with at least 1 to 2 risk factors for stroke according to the CHA_2_DS_2_-VASc (congestive heart failure; hypertension; ages ≥74 years [2 points]; diabetes; stroke, transient ischemic attack, or systemic embolism [2 points]; vascular disease; ages 65-74 years; sex [female]) risk score calculator.^2,3,5,6,7,8,9,10,11,12,13^ However, significant underuse of OAC therapy in patients with AF has been reported in both Europe and the United States.^14,15,16,17,18,19,20,21,22^

In patients with AF who have experienced a stroke, the 90-day risk of early recurrence and long-term recurrence have been reported to be 7.6% and approximately 18%, respectively.^23,24^ However, studies regarding prestroke antithrombotic therapy in patients with AF presenting with stroke are limited and data regarding poststroke antithrombotic therapy in patients with AF and the relationship with the subsequent risk of stroke recurrence are sparse.^21,25^ To address these gaps in knowledge, our aims were to examine prestroke and poststroke antithrombotic therapy in patients with AF admitted with ischemic stroke and to compare long-term outcomes in AF stroke survivors according to poststroke antithrombotic therapy.

## Methods

### Data Sources

The health care system in Denmark is tax financed and without any user charge (except for medication outside the hospital). Data from 4 high-quality nationwide registries were linked using a unique personal identifier number, which all residents are given at birth or immigration. The Danish Civil Registration System holds information about sex, emigration, and date of birth and death.^26^ The Danish National Patient Registry holds information about all inpatient and outpatient hospital contacts, including diagnoses and length of hospital stay.^27^ Diagnoses are registered in terms of *International Classification of Diseases* codes (*International Classification of Diseases, Eighth Revision* until 1994 and *International Statistical Classification of Diseases and Related Health Problems, Tenth Revision* thereafter). The Danish Register of Causes of Death includes information about causes of death.^28^ The Danish National Registry of Medicinal Statistics holds complete information about all filled prescriptions in Denmark but does not hold information about in-hospital prescriptions.^29^ Approval from the research ethics committee system is not required in retrospective registry-based studies in Denmark and a waiver is granted so that informed consent does not have to be obtained. The Danish Data Protection Agency approved use of data for this study.

### Study Patients

We identified all Danish residents with previously diagnosed AF (going back to 1978) who were admitted to a Danish hospital with ischemic stroke (including ischemic stroke, unspecified stroke, and transient ischemic attack [TIA]) from January 1, 2004, to January 31, 2017. According to European AF guidelines, we excluded those younger than 30 years, with valvular AF, and with a CHA_2_DS_2_-VASc score less than 1. The CHA_2_DS_2_-VASc score was calculated using a previously described method.^30^ The remaining patients composed the prestroke population. We also defined a poststroke population that included those who survived a blanking period from hospital admission for stroke until 100 days after discharge. The study index date was set 100 days from stroke discharge to ensure complete information on poststroke antithrombotic treatment since packages of antithrombotic therapy in Denmark include tablets for a maximum of 100 days of treatment. Further, this blanking period ensured exclusion of those who died during their hospital admission for stroke or shortly after. The patients were divided in subgroups according to poststroke antithrombotic therapy and were followed up until June 30, 2017. In a sensitivity analysis, we restricted the patient inclusion to patients with AF admitted with stroke, and therefore excluded those admitted with TIA.

### Antithrombotic Therapy

We divided the prestroke and poststroke study population into 3 study groups according to prestroke and poststroke antithrombotic therapy: OAC therapy (including vitamin K antagonists and non–vitamin K antagonist oral anticoagulants with or without concomitant antiplatelet therapy), antiplatelet therapy alone (aspirin, adenosine diphosphate receptor inhibitors [clopidogrel, prasugrel, and ticagrelor], and dipyridamole), and no antithrombotic therapy. Prestroke antithrombotic therapy was defined as a filled prescription for antithrombotic therapy from 0 to 180 days before hospitalization for stroke. Poststroke antithrombotic therapy was defined as a filled prescription for antithrombotic therapy during the blanking period (0-100 days after stroke discharge). Due to differences in European and US guidelines, we performed a subgroup analysis of prestroke treatment patterns in patients with a prestroke CHA_2_DS_2_-VASc score of 2 or greater. See diagnosis codes and Anatomic Therapeutic Chemical Classification codes in eTable 1 in the Supplement.

### Outcomes

The poststroke population was followed up from the index date until an outcome of interest, end of the study period (June 30, 2017), or emigration, whichever came first. The outcomes included thromboembolic events (ischemic stroke, unspecified stroke, TIA, and embolism or thrombosis in peripheral arteries), bleeding events, and death. Both AF and ischemic stroke were validated in the Danish registries with a positive predictive value of 93% and 97%, respectively, and two-thirds of unspecified strokes were classified as ischemic strokes by raters in a stroke validation study.^31,32^

### Statistical Analysis

Patient demographic characteristics, comorbidities, CHA_2_DS_2_-VASc score, HAS-BLED (hypertension, abnormal renal or liver function, history of stroke, history of bleeding, international normalized ratio [left out due to missing data], ages ≥65 years, drug consumption with antiplatelet agents or nonsteroidal anti-inflammatory drugs, alcohol abuse) risk score for bleeding, and concomitant pharmacotherapy were assessed. The survival probability and cumulative incidence of thromboembolic events according to poststroke antithrombotic therapy were calculated using the Kaplan-Meier estimator and the Aalen-Johansen estimator (incorporating competing risk of death), respectively. The Gray test was used to test for difference in cumulative incidence of thromboembolic events. Crude and adjusted (age, sex, calendar year, comorbidities [ischemic heart disease, peripheral artery disease, heart failure, coagulopathies, chronic kidney disease, chronic obstructive pulmonary disease, alcohol abuse, diabetes, thyroid disease, hypertension, prior bleeding events, pulmonary embolism, and deep venous thrombosis], and concomitant pharmacotherapy [ β-blockers, digoxin, amiodarone, verapamil, and flecainide]) hazard ratios (HRs) were calculated by Cox regression models comparing long-term outcomes according to poststroke antithrombotic therapy. The Cox models were tested for proportional hazard assumptions and linearity of continuous variables. Clinically relevant interactions (sex, calendar year, comorbidities, CHA_2_DS_2_-VASc score, and HAS-BLED score) were assessed. Patient characteristics associated with prestroke and poststroke OAC therapy were assessed by multivariable logistic regression. A *P* value less than .05 was considered statistically significant. All statistical analyses were performed in SAS statistical software version 9.4 (SAS Institute Inc) or R software (R Foundation for Statistical Computing).

## Results

### Prestroke Antithrombotic Therapy

**Figure 1.**
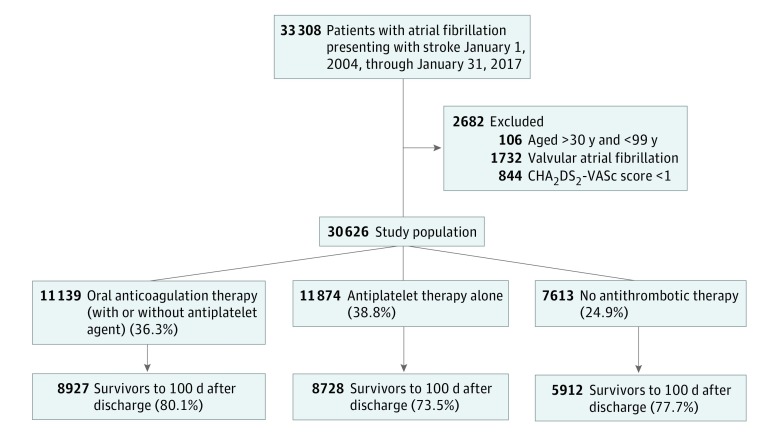
Patient Selection Flowchart CHA_2_DS_2_-VASc indicates risk score for stroke (congestive heart failure; hypertension; ages ≥74 years [2 points]; diabetes; stroke, transient ischemic attack, or systemic embolism [2 points]; vascular disease; ages 65-74 years; sex [female]).

Figure 1 depicts selection of the study population. From January 1, 2004, to January 31, 2017, 33 308 patients with AF presenting with ischemic stroke were admitted to Danish hospitals. After relevant exclusions, the prestroke study population comprised 30 626 patients. Of these, 11 139 patients (36.3%) received OAC therapy, 11 874 (38.8%) received antiplatelet therapy alone, and 7613 (24.9%) did not receive any antithrombotic therapy before their stroke. Time trends in prestroke antithrombotic treatment patterns are depicted in eFigure 1 in the Supplement. The annual percentage of patients receiving OAC therapy before stroke ranged between 30.3% and 31.8% from 2004 to 2010. From 2010, this amount increased, reaching 58.5% in 2017.

The baseline characteristics of the prestroke study population according to prestroke antithrombotic therapy are listed in the Table. Patients receiving OAC therapy were slightly younger and less likely to be female (44.3% female; median age, 79 years [interquartile range {IQR}, 73-85 years]) than those who received antiplatelet therapy (55.0% female; median age, 82 years [IQR, 75-88 years]) or no antithrombotic therapy (53.8% female; median age, 80 years [IQR, 71-86 years]) before their hospital admission for stroke. No differences existed in CHA_2_DS_2_-VASc scores across the different treatment groups. Patients receiving no antithrombotic therapy had a lower estimated HAS-BLED score (median, 2 [IQR, 1-3]) than patients treated with either OAC (median, 3 [IQR, 2-4]) or antiplatelet therapy (median, 3 [IQR, 3-4]). When restricting the prestroke study population to patients with a CHA_2_DS_2_-VASc score of 2 or greater, 10 757 patients (36.5%) received OAC therapy, 11 574 (39.3%) received antiplatelet therapy, and 7106 (24.1%) received no antithrombotic therapy before their stroke diagnosis.

**Table.** Baseline Characteristics at Stroke Hospital Admission According to Prestroke Antithrombotic Therapy in the Prestroke Population

Characteristic	No. (%)			
	Overall (N = 30 626)	OAC Therapy (n = 11 139)^a^	Antiplatelet Therapy Alone (n = 11 874)^b^	No Antithrombotic Therapy (n = 7613)
Demographic				
Age, median (IQR), y	81 (73-86)	79 (73-85)	82 (75-88)	80 (71-86)
Female	15 563 (50.8)	4937 (44.3)	6527 (55.0)	4099 (53.8)
Comorbidities				
Alcohol abuse	1419 (4.6)	411 (3.7)	568 (4.8)	440 (5.8)
Cancer	5597 (18.3)	2167 (19.5)	2045 (17.2)	1385 (18.2)
Coagulopathies	636 (2.1)	339 (3.0)	166 (1.4)	131 (1.7)
Chronic kidney disease	2349 (7.7)	912 (8.2)	961 (8.1)	476 (6.3)
Chronic obstructive pulmonary disease	4143 (13.5)	1578 (14.2)	1680 (14.2)	885 (11.6)
Deep venous thrombosis	1245 (4.1)	596 (5.4)	397 (3.3)	252 (3.3)
Dementia	1677 (5.5)	417 (3.7)	921 (7.8)	339 (4.5)
Diabetes	4836 (15.8)	2089 (18.8)	1925 (16.2)	822 (10.8)
Heart failure	7913 (25.8)	3290 (29.5)	3198 (26.9)	1425 (18.7)
Hypertension	19 363 (63.2)	8090 (72.6)	7850 (66.1)	3423 (45.0)
Ischemic heart disease	11 099 (36.4)	4144 (37.2)	5274 (44.4)	1681 (22.1)
Peripheral artery disease	2734 (8.9)	1073 (9.6)	1252 (10.5)	409 (5.4)
Prior bleeding event	7963 (26.0)	3110 (27.9)	2935 (24.7)	1918 (25.2)
Prior thromboembolic event	11 585 (37.8)	4267 (38.3)	4558 (38.4)	2760 (36.3)
Pulmonary embolism	770 (2.5)	414 (3.7)	191 (1.6)	165 (2.2)
Risk scores, median (IQR)				
CHA_2_DS_2_-VASc^c^	4 (3-5)	4 (3-5)	4 (3-5)	4 (3-5)
HAS-BLED^d^	3 (2-4)	3 (2-4)	3 (3-4)	2 (1-3)
Pharmacotherapy				
Amiodarone	795 (2.6)	425 (3.8)	291 (2.5)	79 (1.0)
β-Blockers	16 056 (52.4)	6920 (62.1)	6422 (54.1)	2714 (35.7)
Digoxin	9580 (31.3)	4426 (39.7)	3677 (31.0)	1477 (19.4)
Flecainide	292 (1.0)	131 (1.2)	101 (0.9)	60 (0.8)
Verapamil	2356 (7.7)	1078 (9.7)	880 (7.4)	398 (5.2)

Abbreviations: IQR, interquartile range; OAC, oral anticoagulation.

^a^ Includes vitamin K antagonists and non–vitamin K OAC with or without antiplatelet agent.

^b^ Includes aspirin, adenosine diphosphate receptor inhibitors (clopidogrel, prasugrel, and ticagrelor), and dipyridamole.

^c^ Risk score for stroke: congestive heart failure; hypertension; ages 74 years or older (2 points); diabetes; stroke, transient ischemic attack, or systemic embolism (2 points); vascular disease; ages 65 to 74 years; sex (female).

^d^ Risk score for bleeding: hypertension, abnormal renal or liver function, history of stroke, history of bleeding, international normalized ratio (left out due to missing data), ages 65 years or older, drug consumption with antiplatelet agents or nonsteroidal anti-inflammatory drugs, alcohol abuse.

### Poststroke Antithrombotic Therapy

Time trends in poststroke antithrombotic therapy are depicted in eFigure 2 in the Supplement. Over the years, OAC treatment rates increased and reached 73.1% during the first 6 months of 2017. From the day of hospital admission for stroke until the index date of 100 days after stroke hospitalization, 19.9%, 26.5%, and 22.3% of patients treated with OAC therapy, antiplatelet therapy, and no antithrombotic therapy, respectively, before their stroke died. eTable 2 in the Supplement illustrates patient characteristics at the index date for the poststroke population grouped by poststroke antithrombotic therapy. Patients treated with OAC therapy following stroke compared with those receiving antiplatelet therapy or no antithrombotic therapy were younger (median [IQR], 77 [70-83] years, 82 [74-87] years, and 80 [74-87] years, respectively), had a lower CHA_2_DS_2_-VASc risk score (median [IQR], 5 [4-6], 6 [5-6], and 5 [4-6], respectively), and had a lower HAS-BLED risk score (median [IQR], 4 [3-4], 4 [4-5], and 3 [3-4], respectively).

### Prestroke to Poststroke Change in Antithrombotic Therapy

**Figure 2.**
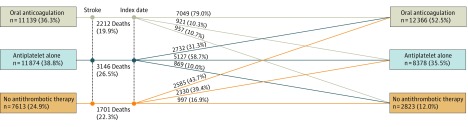
Prestroke to Poststroke Shifts in Antithrombotic Therapy Among Poststroke Study Population

Treatment shifts from before stroke to after stroke among the poststroke population are depicted in Figure 2. The proportion of patients receiving OAC therapy increased from before stroke to after stroke (36.3% vs 52.5%). Of those treated with OAC therapy, antiplatelet therapy, or no antithrombotic therapy before their hospital admission for stroke, 79.0%, 58.7%, and 16.9%, respectively, continued to receive the same treatment after discharge. Following stroke, 31.3% of those receiving antiplatelet therapy alone and 43.7% of those receiving no antithrombotic therapy before stroke shifted to OAC therapy. Yet, 37.5% of patients with stroke did not receive OAC therapy following stroke.

### Long-term Outcomes According to Poststroke Antithrombotic Therapy

**Figure 3.**
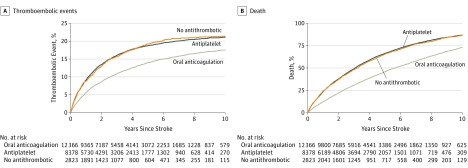
Thromboembolic Events and Death by Poststroke Treatment Group A, Cumulative incidence of thromboembolic events. B, Kaplan-Meier curve for death.

The median follow-up time from the index date to study outcome, death, emigration, or study end was 2.2 years (IQR, 0.8-4.6 years). Figure 3 shows the cumulative incidence of thromboembolic events and deaths according to poststroke antithrombotic therapy. During a maximum of 10 years of follow-up, 17.5%, 21.2%, and 21.5% experienced a new thromboembolic event and 72.7%, 86.4%, and 86.2% died among those treated with OAC therapy, antiplatelet therapy, or no antithrombotic therapy, respectively.

**Figure 4.**
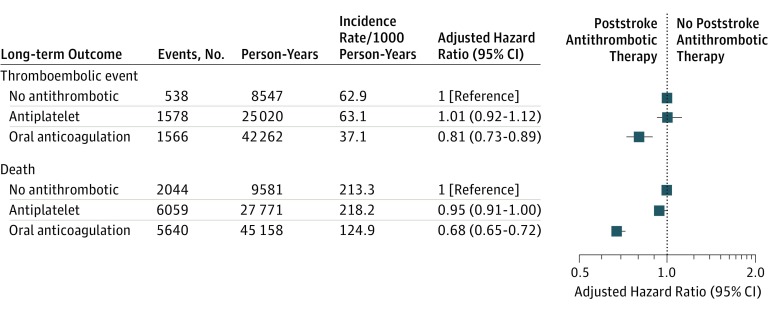
Incidence Rates and Adjusted Hazard Ratios of Long-term Outcomes (Thromboembolic Event and Death) According to Poststroke Treatment Covariates in the adjusted model included age at study start, sex, calendar year, comorbidities (ischemic heart disease, peripheral artery disease, heart failure, prior pulmonary embolism, prior deep venous thrombosis, coagulopathies, chronic kidney disease, prior bleeding event, alcohol abuse, diabetes, hypertension, and chronic obstructive pulmonary disease), and concomitant pharmacotherapy (including β-blockers, digoxin, amiodarone, verapamil, and flecainide).

The long-term risks of stroke (adjusted HR, 0.81; 95% CI, 0.73-0.89) and death (adjusted HR, 0.68; 95% CI, 0.65-0.72) were significantly lower among patients treated with OAC therapy compared with no antithrombotic therapy (Figure 4). In contrast, no significant differences existed for those treated with antiplatelet therapy compared with no antithrombotic therapy (thromboembolic event: adjusted HR, 1.01; 95% CI, 0.92-1.12; death: adjusted HR, 0.95; 95% CI, 0.91-1.00). When comparing the long-term risk of bleeding events according to poststroke antithrombotic therapy, no statistically significant differences were found for OAC therapy (adjusted HR, 0.97; 95% CI, 0.86-1.10) compared with no antithrombotic therapy. The restriction of the study population to patients with a prestroke CHA_2_DS_2_-VASc score of 2 or greater did not change the results (eFigure 3 in the Supplement).

When excluding patients admitted with TIA, the prestroke population consisted of 26 708 patients. With regard to long-term outcomes, the results were similar to those from the main analysis (eFigure 4 in the Supplement).

### Factors Associated With OAC Therapy

Factors associated with prestroke and poststroke OAC therapy are depicted in eFigure 5 and eFigure 6 in the Supplement. For the analysis of the prestroke population, being male, being older than 74 years, and several comorbidities (coagulopathies, deep venous thrombosis, diabetes, heart failure, hypertension, ischemic heart disease, peripheral artery disease, and pulmonary embolism) were associated with OAC therapy, and alcohol abuse, chronic kidney disease, dementia, and prior bleeding events were associated with no OAC therapy. The CHA_2_DS_2_-VASc score was not associated with the prestroke treatment category. For the poststroke analysis, ages 65 to 74 years (compared with ages <65 years), being male, deep venous thrombosis, and hypertension were associated with OAC therapy. Ages greater than 74 years, alcohol abuse, cancer, chronic kidney disease, chronic obstructive pulmonary disease, dementia, ischemic heart disease, and prior bleeding events were associated with no OAC therapy.

## Discussion

We examined prestroke and poststroke antithrombotic therapy in patients with AF with a CHA_2_DS_2_-VASc score of 1 or greater presenting with ischemic stroke, and further related treatment patterns with subsequent long-term outcomes. The study yielded 4 main findings. First, before stroke, almost two-thirds of patients with AF did not receive OAC therapy as recommended by guidelines. Second, after stroke about half of the stroke survivors did not receive OAC therapy. However, prestroke and poststroke treatment rates increased over time. Third, approximately 1 of 5 of those who survived a stroke had a new stroke during follow-up (median, 2.2 years). Fourth, poststroke OAC therapy was associated with a significantly lower risk of a new thromboembolic event without increased risk of bleeding complications compared with antiplatelet therapy or no poststroke antithrombotic therapy. Taken together, these findings suggest substantial opportunities for improvement of both primary and secondary stroke prevention in intermediate- to high-risk patients with AF.

### Prestroke Antithrombotic Therapy

The prestroke use of OAC increased over time, reaching 58.5% in 2017. During the 1990s, several studies showed benefits from OAC therapy (with vitamin K antagonists) instead of aspirin for thromboprophylaxis in patients with AF.^33,34,35,36^ European guidelines changed in 2010, recommending OAC therapy and not aspirin for stroke prevention in patients with AF.^37^ Since then, an increase of OAC therapy has been observed in Denmark among patients with AF,^17^ which is in accordance with this study. Nevertheless, the percentage of eligible patients not receiving any antithrombotic therapy remained approximately 23% even after the new guideline recommendations. Because patients had to pick up the medication at a pharmacy to be registered in a treatment group, bad compliance may have accounted for some part of the patients in the group receiving no antithrombotic therapy. Recently, Xian et al^13^ investigated prestroke antithrombotic therapy in patients with AF presenting with acute ischemic stroke and the association with stroke severity in the Patient-Centered Research Into Outcomes Stroke Patients Prefer and Effectiveness Research study including national stroke data from the United States.^21,38^ In that cohort, approximately 30% received OAC therapy before their stroke diagnosis, 40% received antiplatelet therapy alone, and 30% did not receive any prestroke antithrombotic therapy. These findings are consistent with ours, although Xian and colleagues^13^ were unable to study subsequent treatment patterns and outcomes.

### Poststroke Antithrombotic Therapy

After stroke, 1 in 3 of those who received antiplatelet therapy before stroke shifted to OAC therapy, while almost half of those with no prestroke antithrombotic therapy shifted to OAC therapy. Despite this, almost half of the poststroke population still did not receive OAC therapy. However, treatment rates increased over time. Unfortunately, we had no data on contraindications for OAC therapy and physicians’ considerations when choosing not to prescribe OAC therapy at discharge. In the recent study by Xian et al^13^ using data from the Get With the Guidelines–Stroke program with access to patient files, the most common reasons not to prescribe OAC therapy in patients with AF after stroke were risk of bleeding (16.3%), risk of falls (10.3%), and terminal illness (6.2%). However, 65.8% of the patients did not have a documented reason for no OAC therapy.^21^

In previous studies, we found an association between older age, higher HAS-BLED score, and more comorbidities and no poststroke OAC therapy.^39,40^ This could reflect a caution among physicians in the prescription of OAC therapy for older patients, even though they are at high risk of stroke. However, Appelros and colleagues^39^ recently showed a beneficial effect of OAC therapy for stroke prophylaxis in patients older than 80 years.

### Recurrent Thromboembolic Events

Several studies have examined early recurrence in patients with AF presenting with stroke,^23,41,42^ and a few have looked into the long-term risks.^24,39,43^ Wu et al^24^ found a stroke recurrence rate among patients with AF of 18% during a median follow-up of 2.4 years from stroke hospitalization. This is in accordance with our finding. Patients receiving OAC therapy had a significantly lower risk of a recurrent thromboembolic event compared with those receiving no antithrombotic therapy. Further, no decrease in thromboembolic risk was found for those receiving poststroke antiplatelet therapy. This in part may be related to the possible effectiveness of OAC therapy as secondary stroke prevention in patients with AF. Our results also showed an association between poststroke OAC therapy and lower risk of death. Regarding bleeding events, no significant differences were found across treatment groups. This may be because of residual confounding and confounding by indication, which should be kept in mind when interpreting our results.

### Limitations

This was a retrospective observational study, and no causations can be drawn. Our registries did not include factors such as alcohol consumption or fall tendency. Also, all types of AF were included in the study, and hence also AF occurring secondary to other conditions (eg, postoperative AF). These are factors that may influence the physicians’ choice of antithrombotic therapy. Also, no information on labile international normalized ratio was available for this population, possibly leading to an overestimation of patients receiving adequate OAC therapy. The Danish National Registry of Medicinal Statistics registers redeemed prescriptions, and patients had to pick the medication up to be included in either the OAC or antiplatelet therapy group. Therefore, our results may have been influenced by patient compliance. Moreover, the definition of ischemic stroke included TIA and strokes without classification as hemorrhagic or thrombotic. This may have led to some degree of misclassification. To ensure complete data on antithrombotic therapy, we constructed a blanking period from hospital discharge to 100 days following stroke. During this blanking period several patients died and some of the patients likely had a recurrent thromboembolic event or experienced a bleeding event. This may have led to underestimation of events in the poststroke population. Importantly, this study was based on very high-quality data with nationwide patient inclusion and complete and long-term follow-up ensuring an unbiased selection of study group without loss of follow-up.

## Conclusions

Among patients with AF having an ischemic stroke, OAC therapy rates seemed low both before and after stroke. However, an increase in treatment rates was observed over time. Oral anticoagulation therapy was associated with a significantly lower risk of thromboembolic events without excess risk of bleeding complications compared with antiplatelet therapy and no antithrombotic therapy. This study suggests a substantial opportunity for improving primary and secondary stroke prophylaxis in patients with AF.

## Notes

Supplement. **eTable 1.** Specification of Diagnoses by *ICD-8* and *ICD-10* and Pharmacotherapy by ATC-Codes **eTable 2.** Baseline Characteristics at Stroke Hospital Admission According to Prestroke Antithrombotic Therapy in the Poststroke Population **eFigure 1.** Time Trends in Prestroke Antithrombotic Therapy in Prestroke Population, 2004-2017 **eFigure 2.** Time Trends in Poststroke Antithrombotic Therapy in Poststroke Population, 2004-2017 **eFigure 3.** Adjusted Hazard Ratios of Long-term Outcomes (Thromboembolic Events and Death) According to Poststroke Treatment Group in a Population Restricted to Those With a Prestroke CHA2DS2-VASc ≥2 **eFigure 4.** Adjusted Hazard Ratios of Long-term Outcomes (Thromboembolic Events and Death) According to Poststroke Treatment Group in a Population Restricted to Those Admitted With Ischemic Stroke and Not TIA **eFigure 5.** Factors Associated With Prestroke OAC Therapy **eFigure 6.** Factors Associated With Poststroke OAC Therapy

## References

[1] Lip GYH, Lane DA Stroke prevention in atrial fibrillation: a systematic review. JAMA. 2015;313(19):-. 25988464 10.1001/jama.2015.4369

[2] Freedman B, Potpara TS, Lip GYH Stroke prevention in atrial fibrillation. Lancet. 2016;388(10046):806-817. 27560276 10.1016/S0140-6736(16)31257-0

[3] Kirchhof P, Benussi S, Kotecha D, ; ESC Scientific Document Group 2016 ESC Guidelines for the management of atrial fibrillation developed in collaboration with EACTS. Eur Heart J. 2016;37(38):2893-2962. 27567408 10.1093/eurheartj/ehw210

[4] Camm AJ, Lip GYH, De Caterina R, ; ESC Committee for Practice Guidelines-CPG; Document Reviewers 2012 focused update of the ESC Guidelines for the management of atrial fibrillation: an update of the 2010 ESC Guidelines for the management of atrial fibrillation—developed with the special contribution of the European Heart Rhythm Association. Europace. 2012;14(10):1385-1413. 22923145 10.1093/europace/eus305

[5] Ruff CT, Giugliano RP, Braunwald E, Comparison of the efficacy and safety of new oral anticoagulants with warfarin in patients with atrial fibrillation: a meta-analysis of randomised trials. Lancet. 2014;383(9921):955-962. 24315724 10.1016/S0140-6736(13)62343-0

[6] Hart RG, Pearce LA, Aguilar MI Meta-analysis: antithrombotic therapy to prevent stroke in patients who have nonvalvular atrial fibrillation. Ann Intern Med. 2007;146(12):857-867. 17577005 10.7326/0003-4819-146-12-200706190-00007

[7] Connolly SJ, Ezekowitz MD, Yusuf S, ; RE-LY Steering Committee and Investigators Dabigatran versus warfarin in patients with atrial fibrillation. N Engl J Med. 2009;361(12):1139-1151. 19717844 10.1056/NEJMoa0905561

[8] Patel MR, Mahaffey KW, Garg J, ; ROCKET AF Investigators Rivaroxaban versus warfarin in nonvalvular atrial fibrillation. N Engl J Med. 2011;365(10):883-891. 21830957 10.1056/NEJMoa1009638

[9] Granger CB, Alexander JH, McMurray JJV, ; ARISTOTLE Committees and Investigators Apixaban versus warfarin in patients with atrial fibrillation. N Engl J Med. 2011;365(11):981-992. 21870978 10.1056/NEJMoa1107039

[10] Giugliano RP, Ruff CT, Braunwald E, ; ENGAGE AF-TIMI 48 Investigators Edoxaban versus warfarin in patients with atrial fibrillation. N Engl J Med. 2013;369(22):2093-2104. 24251359 10.1056/NEJMoa1310907

[11] Meschia JF, Bushnell C, Boden-Albala B, ; American Heart Association Stroke Council; Council on Cardiovascular and Stroke Nursing; Council on Clinical Cardiology; Council on Functional Genomics and Translational Biology; Council on Hypertension Guidelines for the primary prevention of stroke: a statement for healthcare professionals from the American Heart Association/American Stroke Association. Stroke. 2014;45(12):3754-3832. 25355838 10.1161/STR.0000000000000046 PMC5020564

[12] Kernan WN, Ovbiagele B, Black HR, ; American Heart Association Stroke Council, Council on Cardiovascular and Stroke Nursing, Council on Clinical Cardiology, and Council on Peripheral Vascular Disease Guidelines for the prevention of stroke in patients with stroke and transient ischemic attack: a guideline for healthcare professionals from the American Heart Association/American Stroke Association. Stroke. 2014;45(7):2160-2236. 24788967 10.1161/STR.0000000000000024

[13] Xian Y, Wu J, O’Brien EC, Real world effectiveness of warfarin among ischemic stroke patients with atrial fibrillation: observational analysis from Patient-Centered Research Into Outcomes Stroke Patients Prefer and Effectiveness Research (PROSPER) study. BMJ. 2015;351:h3786. 26232340 10.1136/bmj.h3786 PMC4521370

[14] Brass LM, Krumholz HM, Scinto JM, Radford M Warfarin use among patients with atrial fibrillation. Stroke. 1997;28(12):2382-2389. 9412618 10.1161/01.str.28.12.2382

[15] Wilke T, Groth A, Mueller S, Oral anticoagulation use by patients with atrial fibrillation in Germany: adherence to guidelines, causes of anticoagulation under-use and its clinical outcomes, based on claims-data of 183,448 patients. Thromb Haemost. 2012;107(6):1053-1065. 22398417 10.1160/TH11-11-0768

[16] Hsu JC, Maddox TM, Kennedy KF, Oral anticoagulant therapy prescription in patients with atrial fibrillation across the spectrum of stroke risk: insights from the NCDR PINNACLE registry. JAMA Cardiol. 2016;1(1):55-62. 27437655 10.1001/jamacardio.2015.0374

[17] Gadsbøll K, Staerk L, Fosbøl EL, Increased use of oral anticoagulants in patients with atrial fibrillation: temporal trends from 2005 to 2015 in Denmark. Eur Heart J. 2017;38(12):899-906. 28110293 10.1093/eurheartj/ehw658

[18] Dlott JS, George RA, Huang X, National assessment of warfarin anticoagulation therapy for stroke prevention in atrial fibrillation. Circulation. 2014;129(13):1407-1414. 24493817 10.1161/CIRCULATIONAHA.113.002601

[19] Waldo AL, Becker RC, Tapson VF, Colgan KJ; NABOR Steering Committee Hospitalized patients with atrial fibrillation and a high risk of stroke are not being provided with adequate anticoagulation. J Am Coll Cardiol. 2005;46(9):1729-1736. 16256877 10.1016/j.jacc.2005.06.077

[20] Gladstone DJ, Bui E, Fang J, Potentially preventable strokes in high-risk patients with atrial fibrillation who are not adequately anticoagulated. Stroke. 2009;40(1):235-240. 18757287 10.1161/STROKEAHA.108.516344

[21] Xian Y, O’Brien EC, Liang L, Association of preceding antithrombotic treatment with acute ischemic stroke severity and in-hospital outcomes among patients with atrial fibrillation. JAMA. 2017;317(10):1057-1067. 28291892 10.1001/jama.2017.1371

[22] Hsu JC, Maddox TM, Kennedy K, Aspirin instead of oral anticoagulant prescription in atrial fibrillation patients at risk for stroke. J Am Coll Cardiol. 2016;67(25):2913-2923. 27339487 10.1016/j.jacc.2016.03.581

[23] Paciaroni M, Agnelli G, Falocci N, Early recurrence and cerebral bleeding in patients with acute ischemic stroke and atrial fibrillation: effect of anticoagulation and its timing: the RAF study. Stroke. 2015;46(8):2175-2182. 26130094 10.1161/STROKEAHA.115.008891

[24] Wu Y-L, Saver JL, Chen P-C, Effect of statin use on clinical outcomes in ischemic stroke patients with atrial fibrillation. Medicine (Baltimore). 2017;96(5):e5918. 28151869 10.1097/MD.0000000000005918 PMC5293432

[25] Björck S, Palaszewski B, Friberg L, Bergfeldt L Atrial fibrillation, stroke risk, and warfarin therapy revisited: a population-based study. Stroke. 2013;44(11):3103-3108. 23982711 10.1161/STROKEAHA.113.002329

[26] Pedersen CB The Danish Civil Registration System. Scand J Public Health. 2011;39(7)(suppl):22-25. 21775345 10.1177/1403494810387965

[27] Lynge E, Sandegaard JL, Rebolj M The Danish national patient register. Scand J Public Health. 2011;39(7)(suppl):30-33. 21775347 10.1177/1403494811401482

[28] Helweg-Larsen K The Danish register of causes of death. Scand J Public Health. 2011;39(7)(suppl):26-29. 21775346 10.1177/1403494811399958

[29] Kildemoes HW, Sørensen HT, Hallas J The Danish national prescription registry. Scand J Public Health. 2011;39(7)(suppl):38-41. 21775349 10.1177/1403494810394717

[30] Olesen JB, Lip GYH, Hansen ML, Validation of risk stratification schemes for predicting stroke and thromboembolism in patients with atrial fibrillation: nationwide cohort study. BMJ. 2011;342:d124. 21282258 10.1136/bmj.d124 PMC3031123

[31] Rix TA, Riahi S, Overvad K, Lundbye-Christensen S, Schmidt EB, Joensen AM Validity of the diagnoses atrial fibrillation and atrial flutter in a Danish patient registry. Scand Cardiovasc J. 2012;46(3):149-153. 22397620 10.3109/14017431.2012.673728

[32] Krarup L-H, Boysen G, Janjua H, Prescott E, Truelsen T Validity of stroke diagnoses in a national register of patients. Neuroepidemiology. 2007;28(3):150-154. 17478969 10.1159/000102143

[33] Petersen P, Boysen G, Godtfredsen J, Andersen ED, Andersen B Placebo-controlled, randomised trial of warfarin and aspirin for prevention of thromboembolic complications in chronic atrial fibrillation: the Copenhagen AFASAK study. Lancet. 1989;1(8631):175-179. 2563096 10.1016/s0140-6736(89)91200-2

[34] Singer DE, Hughes RA, Gress DR, ; Boston Area Anticoagulation Trial for Atrial Fibrillation Investigators The effect of low-dose warfarin on the risk of stroke in patients with nonrheumatic atrial fibrillation. N Engl J Med. 1990;323(22):1505-1511. 2233931 10.1056/NEJM199011293232201

[35] Ezekowitz MD, Bridgers SL, James KE, ; Veterans Affairs Stroke Prevention in Nonrheumatic Atrial Fibrillation Investigators Warfarin in the prevention of stroke associated with nonrheumatic atrial fibrillation. N Engl J Med. 1992;327(20):1406-1412. 1406859 10.1056/NEJM199211123272002

[36] Go AS, Hylek EM, Borowsky LH, Phillips KA, Selby JV, Singer DE Warfarin use among ambulatory patients with nonvalvular atrial fibrillation: the Anticoagulation and Risk Factors in Atrial Fibrillation (ATRIA) study. Ann Intern Med. 1999;131(12):927-934. 10610643 10.7326/0003-4819-131-12-199912210-00004

[37] Camm AJ, Kirchhof P, Lip GY, ; ESC Committee for Practice Guidelines Guidelines for the management of atrial fibrillation: the Task Force for the Management of Atrial Fibrillation of the European Society of Cardiology (ESC). Europace. 2010;12(10):1360-1420. 20876603 10.1093/europace/euq350

[38] Xian Y, O’Brien EC, Fonarow GC, Patient-Centered Research Into Outcomes Stroke Patients Prefer and Effectiveness Research: implementing the patient-driven research paradigm to aid decision making in stroke care. Am Heart J. 2015;170(1):36-45, 45.e1-45.e11. 26093862 10.1016/j.ahj.2015.04.008

[39] Appelros P, Farahmand B, Terént A, Åsberg S To treat or not to treat: anticoagulants as secondary preventives to the oldest old with atrial fibrillation. Stroke. 2017;48(6):1617-1623. 28487335 10.1161/STROKEAHA.117.016902

[40] Yiin GSC, Howard DPJ, Paul NLM, ; Oxford Vascular Study Age-specific incidence, outcome, cost, and projected future burden of atrial fibrillation-related embolic vascular events: a population-based study. Circulation. 2014;130(15):1236-1244. 25208551 10.1161/CIRCULATIONAHA.114.010942 PMC5384634

[41] Hart RG, Coull BM, Hart D Early recurrent embolism associated with nonvalvular atrial fibrillation: a retrospective study. Stroke. 1983;14(5):688-693. 6658950 10.1161/01.str.14.5.688

[42] Kelley RE, Berger JR, Alter M, Kovacs AG Cerebral ischemia and atrial fibrillation: prospective study. Neurology. 1984;34(10):1285-1291. 6541300 10.1212/wnl.34.10.1285

[43] McGrath ER, Kapral MK, Fang J, ; Investigators of the Ontario Stroke Registry Antithrombotic therapy after acute ischemic stroke in patients with atrial fibrillation. Stroke. 2014;45(12):3637-3642. 25378422 10.1161/STROKEAHA.114.006929

