# Supplementary material for: Prestroke and Poststroke Antithrombotic Therapy in Patients With Atrial Fibrillation: Results From a Nationwide Cohort
Source: JAMA Netw Open. 2018 May 18;1(1):e180171. doi: 10.1001/jamanetworkopen.2018.0171 (PMC6324317; doi:10.1001/jamanetworkopen.2018.0171)
Supplement: Supplement. — eTable 1. Specification of Diagnoses by ICD-8 and ICD-10 and Pharmacotherapy by ATC-Codes eTable 2. Baseline Characteristics at Stroke Hospital Admission According to Prestroke Antithrombotic Therapy in the Poststroke Population eFigure 1. Time Trends in Prestroke Antithrombotic Therapy in Prestroke Population, 2004-2017 eFigure 2. Time Trends in Poststroke Antithrombotic Therapy in Poststroke Population, 2004-2017 eFigure 3. Adjusted Hazard Ratios of Long-term Outcomes (Thromboembolic Events and Death) According to Poststroke Treatment Group in a Population Restricted to Those With a Prestroke CHA2DS2-VASc ≥2 eFigure 4. Adjusted Hazard Ratios of Long-term Outcomes (Thromboembolic Events and Death) According to Poststroke Treatment Group in a Population Restricted to Those Admitted With Ischemic Stroke and Not TIA eFigure 5. Factors Associated With Prestroke OAC Therapy eFigure 6. Factors Associated With Poststroke OAC Therapy [file jamanetwopen-1-e180171-s001.pdf]

Supplementary Online Content

Gundlund A, Xian Y, Peterson ED, et al. Prestroke and poststroke antithrombotic therapy in patients with atrial fibrillation: results from a nationwide cohort. *JAMA Netw Open*. 2018;1(1):e180171.
doi:10.1001jamanetworkopen.2018.0171

eTable 1. Specification of Diagnoses by *ICD-8* and *ICD-10* and Pharmacotherapy by ATC-Codes
eTable 2. Baseline Characteristics at Stroke Hospital Admission According to Prestroke Antithrombotic Therapy in the Poststroke Population
eFigure 1. Time Trends in Prestroke Antithrombotic Therapy in Prestroke Population, 2004-2017
eFigure 2. Time Trends in Poststroke Antithrombotic Therapy in Poststroke Population, 2004-2017
eFigure 3. Adjusted Hazard Ratios of Long-term Outcomes (Thromboembolic Events and Death) According to Poststroke Treatment Group in a Population Restricted to Those With a Prestroke CHA2DS2-VASc≥2
eFigure 4. Adjusted Hazard Ratios of Long-term Outcomes (Thromboembolic Events and Death) According to Poststroke Treatment Group in a Population Restricted to Those Admitted With Ischemic Stroke and Not TIA
eFigure 5. Factors Associated With Prestroke OAC Therapy
eFigure 6. Factors Associated With Poststroke OAC Therapy

This supplementary material has been provided by the authors to give readers additional information about their work.

eTable 1. Specification of Diagnoses by *ICD-8* and *ICD-10* and Pharmacotherapy by ATC-Codes

**Diagnoses**	**ICD-10 codes (and ICD-8 codes) and ATC-codes**
Atrial fibrillation	I48<br>ICD-8: 42793, 42794
Alcohol abuse	F10, K70, E52, T51, K860, E244, G312, I426, O354, Z714, Z721, G621, G721, K292, L278A<br>ATC: N07BB
Bleeding	Heart: I312<br>Urine: N02, R31<br>Airways: R04<br>Eye: H313, H356, H431, H450, H052A<br>Gastro intestinal: K228F, K250, K252, K254, K256, K260, K262, K264, K266, K270, K272, K274, K276, K280, K282, K284, K286, K298A, K625, K638B, K638C, K661, K838F, K868G, K920, K921, K922, I850, I864A<br>Intra-dural bleeds (excl. hemorrhagic stroke): S064, S065, S066, G951A<br>Retro-peritoneal: S368D<br>Thorax: J942<br>Anemia due to bleeding: D500, D62
Cancer	All C-diagnoses
Chronic kidney disease	E102, E112, E132, E142, I120, M321B, N02, N03, N04, N05, N06, N07, N08, N11, N12, N14, N18, N19, N26, N158, N159, N160, N162, N163, N164, N168 Q61
Chronic obstructive pulmonary disease	J42, J43, J44
Coagulopathies	D66, D67, D68, D69
Deep venous thrombosis	I801, I802, I803, I808, I809, I821, I822, I823, I828, I829
Dementia	F000-F002, F009-FF013, F018, F019, F051, F01-F03, F1073, F1173, F1273, F1373, F1473, F1573, F1673, F1873, F1973, F1474
Diabetes	ATC: A10
Heart failure	I42, I50, I110, J81,
Hypertension	Usage of combination of at least two of the seven different drugs classes at the same time:<br>1. Non-Loop diuretics: C02L, C02DA, C07B, C07D, C09XA52, C03A, C03EA, C03B, C03X, C07C, C08G, C09BA, C09DA, C03D, C03E, C03EB

© 2018 Gundlund A et al. *JAMA Network Open.*

	2. Loop diuretics C03C, C03EB<br>3. Antiadrenergic agents: C02A, C02B, C02C<br>4. Beta-blockers: C07A, C07B, C07C, C07D, C07F<br>5. Vasodilators: C02DB, C02DD, C02DG<br>6. Calcium channel blockers: C08, C09BB, C09DB<br>7. Renin angiotensin receptor blockers: C09AA, C09BA, C09BB, C09CA, C09DA, C09DB, C09XA02, C09XA52
Ischemic heart disease	I21, I22, I23, I24, I25
Peripheral artery disease	I70
Bleeding	D50.0, H31.3, H35.6, H43.1, H45.0, G95.1A, I31.2, I85.0, I86.4A, H05.2A, J94.2, K22.8F, K25.0, K25.2, K25.4, K25.6, K26.0, K26.2, K26.4, K26.6, K27.0 K27.2, K27.4, K27.6, K28.0, K28.2, K28.4, K28.6, K29.8A, K62.5, K63.8B, K63.8C, K66.1, K83.8F, K86.8G, K92.0-K92.2, S06.4, S06.6, S36.8D
Pulmonary embolism	I26
Ischemic stroke	G45.8, G45.9, I63, I64
Thromboembolic event	Stroke and I74
**Pharmacotherapy**	**ATC-codes**
Amiodarone	C01BD01
Anticoagulation therapy	Non-vitamin K antagonist oral anticoagulants: B01AE07, B01AF01, B01AF02, B01F03<br>Vitamin K antagonists: B01AA03, B01AA04
Antiplatelet therapy	Aspirin: B01AC06<br>ADP-receptor blockers: B01AC22, B01AC24<br>Clopidogrel: B01AC04<br>Dipyridamole: B01AC07
Beta-blockers	C07A, C07B, C07C, C07D, C07F
Digoxin	C01AA
Flecainide	C01BC
Verapamil	C08DA

© 2018 Gundlund A et al. *JAMA Network Open*.

eTable 2. Baseline Characteristics at Stroke Hospital Admission According to Prestroke Antithrombotic Therapy in the Poststroke Population

	Overall N=23,567 (100%)	OAC therapy[a] N=12,366 (52.5%)	Antiplatelet therapy alone[b] N=8378 (35.6%)	No antithrombotic therapy N=2823 (12.0%)
**Demographics**				
Age, median years (IQR)	79 (72-85)	77 (70-83)	82 (74-87)	80 (74-87)
Female, n (%)	11,444 (48.6)	5592 (45.2)	4502 (53.7)	1350 (47.8)
**Comorbidities, n (%)**				
Alcohol abuse	1269 (5.4)	535 (4.3)	533 (6.4)	201 (7.1)
Cancer	4358 (18.5)	2187 (17.7)	1594 (19.0)	577 (20.4)
Coagulopathies	559 (2.4)	306 (2.5)	173 (2.1)	80 (2.8)
Chronic kidney disease	1940 (8.2)	896 (7.3)	787 (9.4)	257 (9.1)
COPD[c]	3361 (14.3)	1641 (13.3)	1319 (15.7)	401 (14.2)
Deep venous thrombosis	997 (4.2)	583 (4.7)	293 (3.5)	121 (4.3)
Dementia	1555 (6.6)	468 (3.8)	844 (10.1)	243 (8.6)
Diabetes	3854 (16.4)	2038 (16.5)	1395 (16.7)	421 (14.9)
Heart failure	6325 (26.8)	3203 (25.9)	2348 (28.0)	774 (27.4)
Hypertension	16,140 (68.5)	8871 (71.7)	5581 (66.6)	1688 (59.8)
Ischemic heart disease	9194 (39.0)	4411 (35.7)	3710 (44.3)	1073 (38.0)
Peripheral artery disease	2113 (9.0)	995 (8.1)	846 (10.1)	272 (9.6)
Prior bleeding event	6563 (27.9)	3032 (24.5)	2426 (29.0)	1105 (39.1)
Pulmonary embolism	635 (2.7)	373 (3.0)	176 (2.1)	86 (3.1)
**Risk scores**				
CHA_2DS_2-VASc[d], median (IQR)	5 (4-6)	5 (4-6)	6 (5-6)	5 (4-6)
HAS-BLED[e], median (IQR)	4 (3-4)	3 (3-4)	4 (4-5)	3 (3-4)
**Pharmacotherapy**				
Amiodarone	843 (3.6)	506 (4.1)	242 (2.9)	95 (3.4)
Beta blockers	13,740	8077	4301 (51.3)	1362 (48.3)

© 2018 Gundlund A et al. *JAMA Network Open.*

	(58.3)	(65.3)		
Digoxin	8405 (35.7)	4623 (37.4)	2756 (32.9)	1026 (36.3)
Flecainide	246 (1.0)	167 (1.4)	60 (0.7)	19 (0.7)
Verapamil	1791 (7.6)	976 (7.9)	611 (7.3)	204 (7.2)

[a]Oral anticoagulation therapy: Including vitamin K antagonists and non-vitamin K oral anticoagulants with or without antiplatelet agent. [b]Including aspirin, ADP-receptor inhibitors (clopidogrel, prasugrel, ticagrelor), and dipyridamole. [c]Chronic Obstructive Pulmonary disease. [d]Risk score for stroke: congestive heart failure, hypertension, age>74 years (2 points), diabetes, stroke/TIA/systemic embolism (2 points), vascular disease, age 65-74 years, sex category (female). [e]Risk score for bleeding: hypertension, abnormal renal/liver function, history of stroke, history of bleeding, INR (left out due to missing data), age>65 years, drug consumption with antiplatelet agents/non-steroidal inflammatory drugs, alcohol abuse.

© 2018 Gundlund A et al. *JAMA Network Open*.

eFigure 1. Time Trends in Prestroke Antithrombotic Therapy in Prestroke Population, 2004-2017

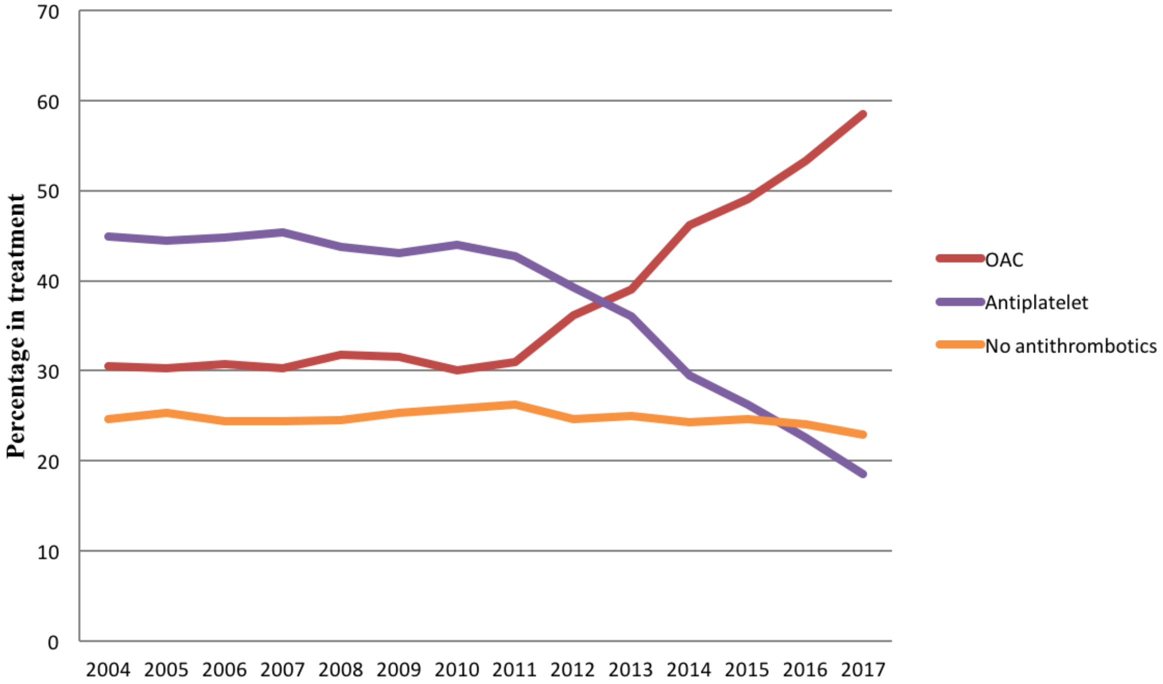

*October 2010: New ESC guidelines for management of AF.

© 2018 Gundlund A et al. *JAMA Network Open*.

eFigure 2. Time Trends in Poststroke Antithrombotic Therapy in Poststroke Population, 2004-2017

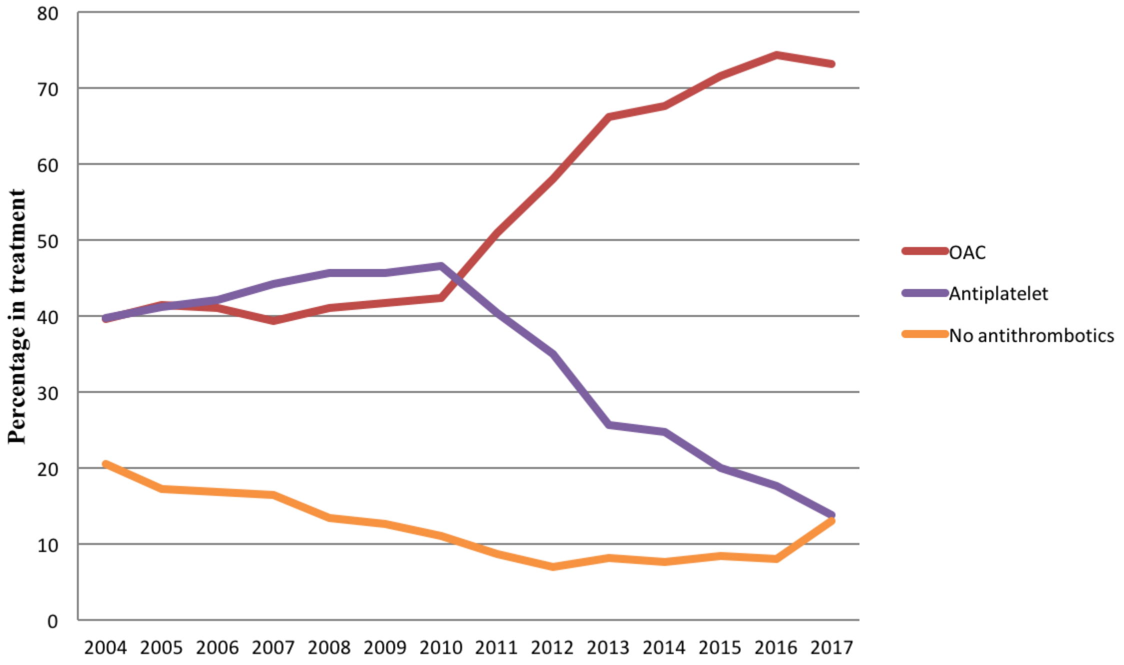

*October 2010: New ESC guidelines for management of AF. AF=atrial fibrillation.

© 2018 Gundlund A et al. *JAMA Network Open*.

eFigure 3. Adjusted Hazard Ratios of Long-term Outcomes (Thromboembolic Events and Death) According to Poststroke Treatment Group in a Population Restricted to Those With a Prestroke CHA2DS2-VASc≥2

Groups	Adjusted HR [95% CI]
Thromboembolic event	
No antithrombotics	Ref.
Antiplatelet	1.01 (0.92-1.12)
OAC	0.81 (0.73-0.90)
Death	
No antithrombotics	Ref.
Antiplatelet	0.96 (0.91-1.01)
OAC	0.69 (0.65-0.72)

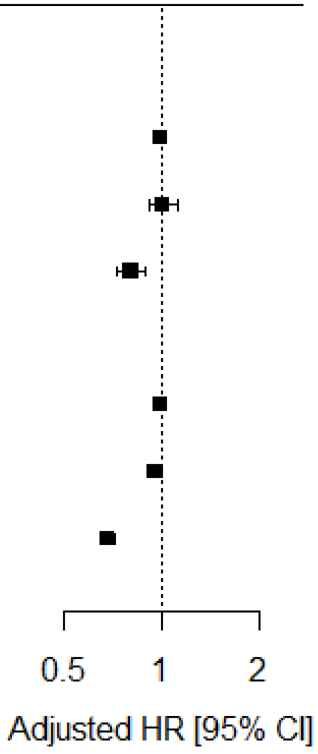

HR=Hazard ratio, 95% CI= 95% confidence limits.

© 2018 Gundlund A et al. *JAMA Network Open.*

eFigure 4. Adjusted Hazard Ratios of Long-term Outcomes (Thromboembolic Events and Death) According to Poststroke Treatment Group in a Population Restricted to Those Admitted With Ischemic Stroke and Not TIA

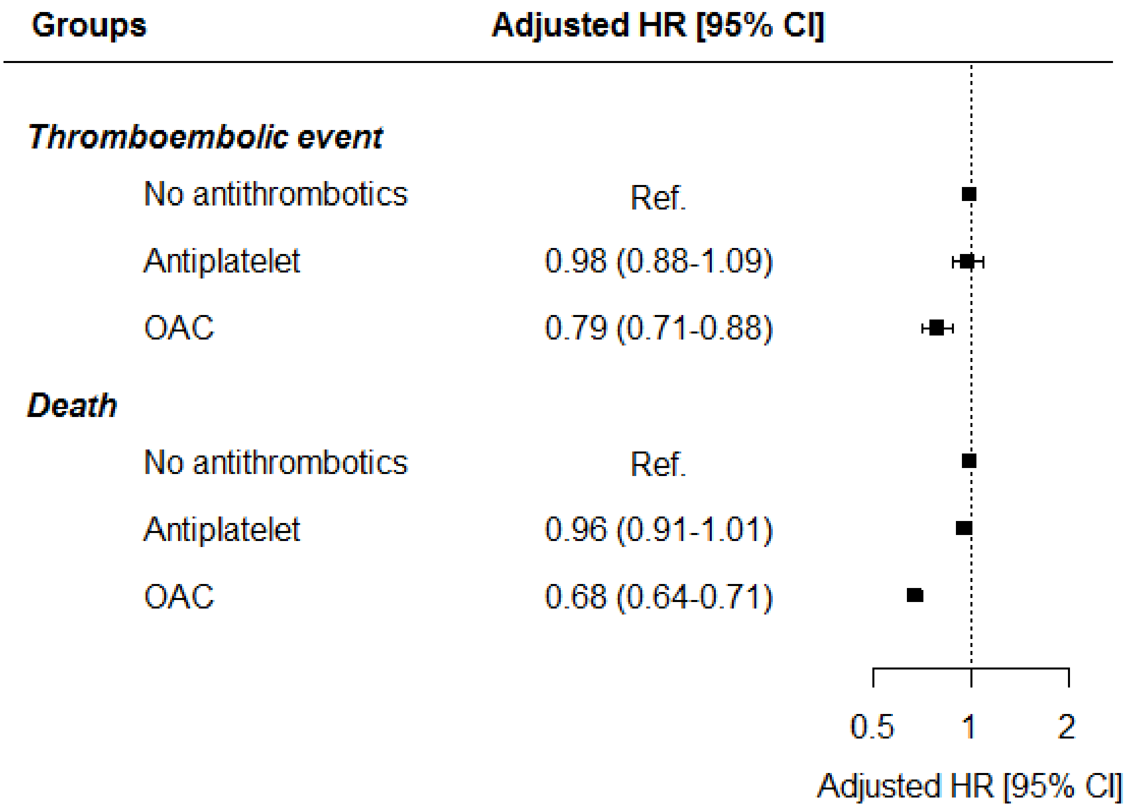

HR= Hazard ratio, 95% CI= 95% confidence limits, TIA=transient ischemic attack.

© 2018 Gundlund A et al. *JAMA Network Open*.

eFigure 5. Factors Associated With Prestroke OAC Therapy

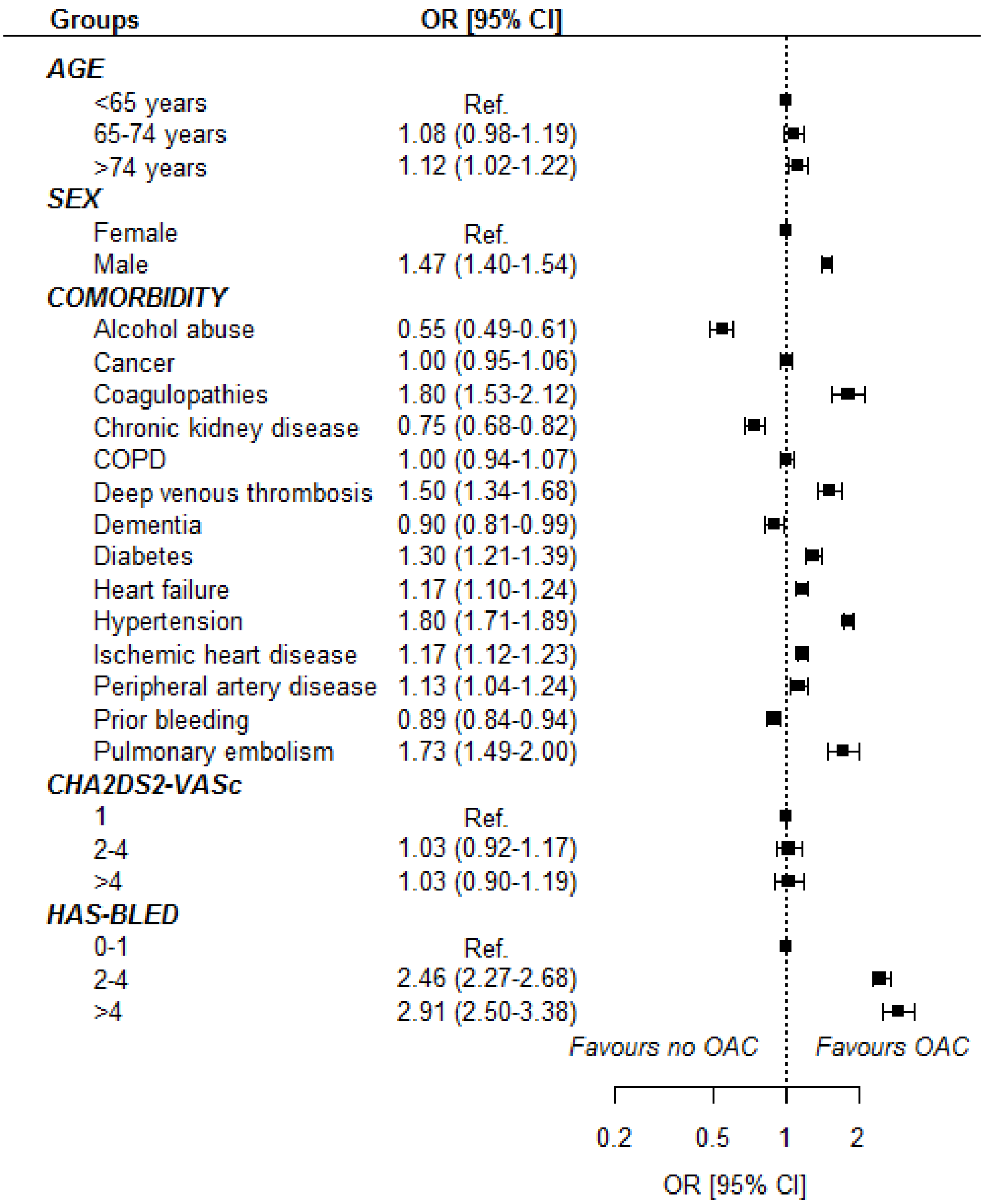

COPD=chronic obstructive pulmonary disease. CI=confidence interval. OAC=oral anticoagulation. OR=Odds ratio.

© 2018 Gundlund A et al. *JAMA Network Open*.

eFigure 6. Factors Associated With Poststroke OAC Therapy

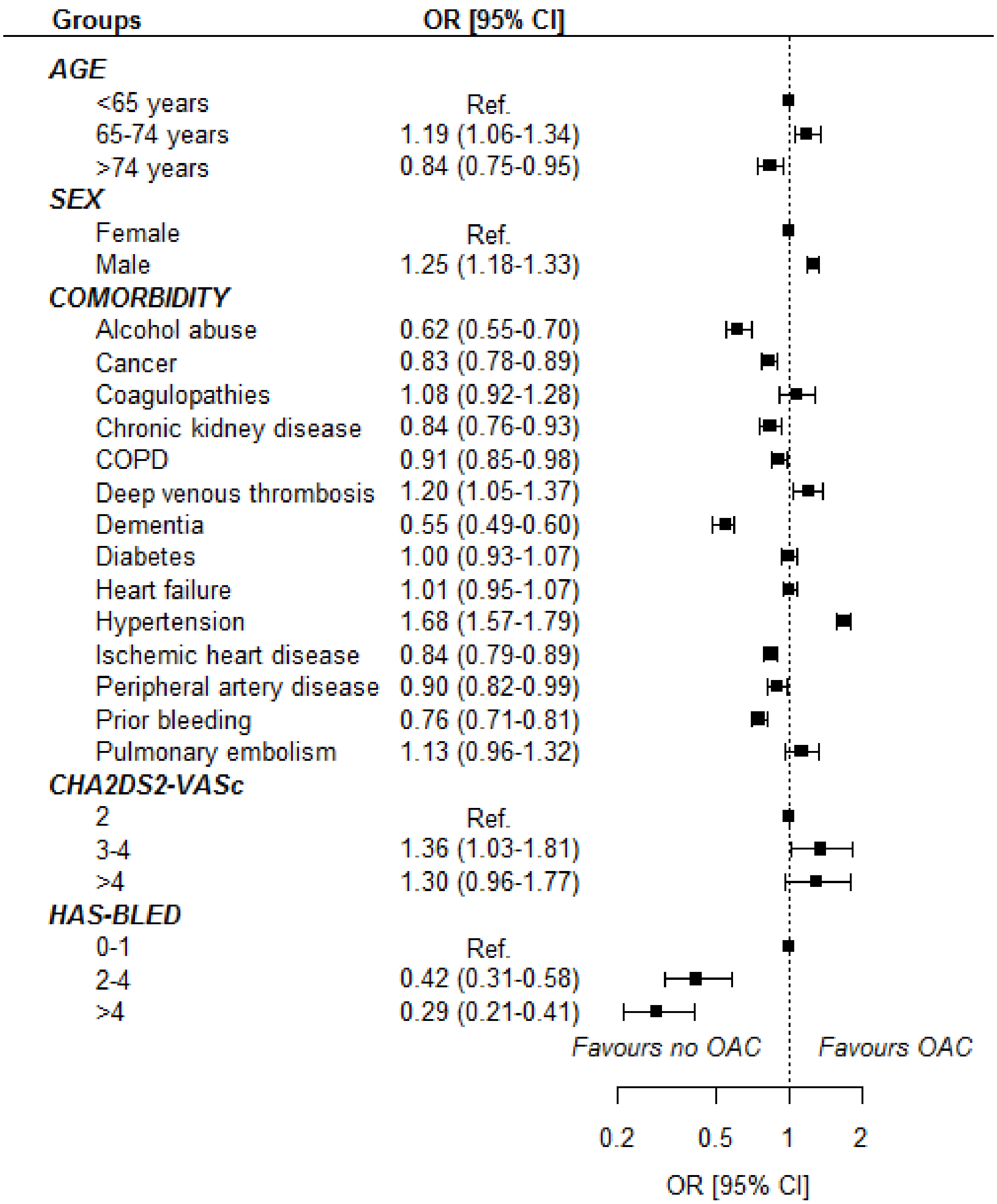

COPD=chronic obstructive pulmonary disease. CI=confidence interval. OAC=oral anticoagulation. OR=Odds ratio.

© 2018 Gundlund A et al. *JAMA Network Open*.
